# Rapidly Progressing Moyamoya Syndrome Secondary to Meningovascular Neurosyphilis and Acquired Immunodeficiency Syndrome

**DOI:** 10.7759/cureus.16123

**Published:** 2021-07-02

**Authors:** Branden C Wilson, Matthew Bear, Aswin Srinivasan, Khulood Rizvi, Samer Elfallal, Xiang Fang, Mohamad Ezzeldin

**Affiliations:** 1 Internal Medicine, HCA Houston Kingwood/University of Houston College of Medicine, Kingwood, USA; 2 Infectious Disease, HCA Houston Kingwood/University of Houston College of Medicine, Kingwood, USA; 3 Neurosurgery, HCA Houston Kingwood/University of Houston College of Medicine, Kingwood, USA; 4 Neurology, University of Texas Medical Branch, Galveston, USA; 5 Neuroendovascular Surgery, HCA Houston Kingwood/University of Houston College of Medicine, Kingwood, USA

**Keywords:** moyamoya syndrome, vasculitis, neurosyphilis, human immunodeficiency virus, stroke

## Abstract

Moyamoya syndrome is a chronic and progressive narrowing of the arteries in the brain caused by different mechanisms than the genetic mutation that leads to moyamoya disease. It is characterized by the narrowing and/or closing of the carotid artery with a collateral circulation development around the blocked vessels to compensate for the ischemia. In this report, we present a unique case of moyamoya syndrome that developed over the course of a few months in a patient with new-onset strokes and seizures in the setting of late diagnosis of neurosyphilis and acquired immunodeficiency syndrome (AIDS). To our knowledge, moyamoya syndrome secondary to coinfection with AIDS and meningovascular neurosyphilis has only been reported once in the literature.

## Introduction

Syphilis is a sexually transmitted disease caused by *Treponema pallidum*. This disease follows a course of four clinical stages: primary, secondary, latent, and tertiary. Neurosyphilis refers to the involvement of the central nervous system and can occur at any stage if untreated. It is more commonly seen in the human immunodeficiency virus (HIV) population [1,2]. Neurological symptoms can be the first and sole manifestation of the disease [2]. We present a unique case of new-onset strokes and seizures in a patient with a late diagnosis of acquired immunodeficiency syndrome (AIDS) and neurosyphilis with rapidly progressive moyamoya syndrome.

## Case presentation

**Figure 1:**
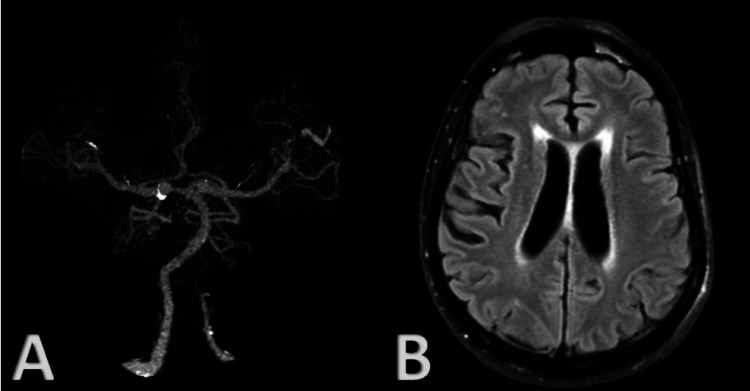
(A) 3D reconstruction of CTA head demonstrating normal intracranial anterior and posterior circulation. (B) Axial FLAIR sequence MRI brain demonstrating chronic microvascular changes particularly in the periatrial region. 3D: three-dimensional; CTA: computed tomography angiography; FLAIR: fluid-attenuated inversion recovery; MRI: magnetic resonance imaging

A 64-year-old Caucasian male with a past medical history of smoking and hypertension presented to the emergency department with acute onset right-sided hemiplegia and dysphasia, which resolved after tissue plasminogen activator (tPA) treatment. Computed tomography angiography (CTA) of the head showed normal intracranial anterior and posterior circulation (Figure 1A). Magnetic resonance imaging (MRI) of the brain demonstrated only chronic microvascular changes in the periatrial region (Figure 1B). The patient was discharged home with no focal neurological deficits on daily aspirin.

**Figure 2:**
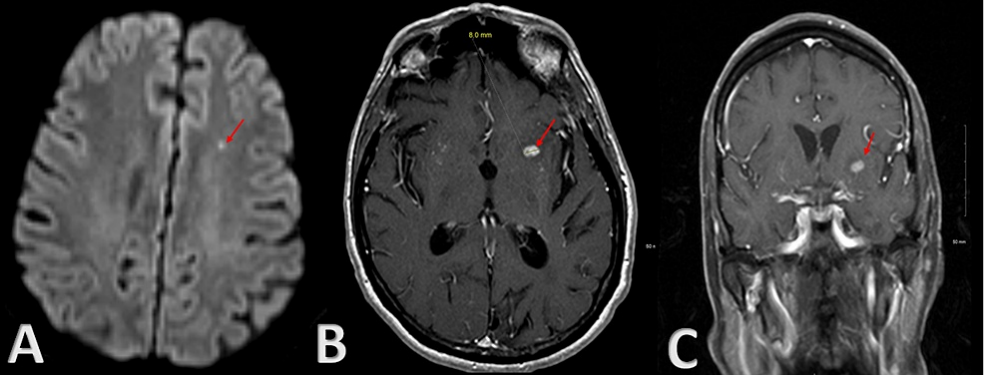
(A) Axial DWI sequence MRI brain demonstrating small acute ischemic infarct in the left precentral gyrus as well as a 2 cm mass lesion in the left basal ganglia (red arrow). (B) Axial T1 sequence MRI brain with contrast demonstrating a 2 cm mass lesion in the left basal ganglia (red arrow). (C) Coronal T1 sequence MRI brain with contrast demonstrating a 2 cm mass lesion in the left basal ganglia (red arrow). DWI: diffusion-weighted imaging; MRI: magnetic resonance imaging

**Figure 3:**
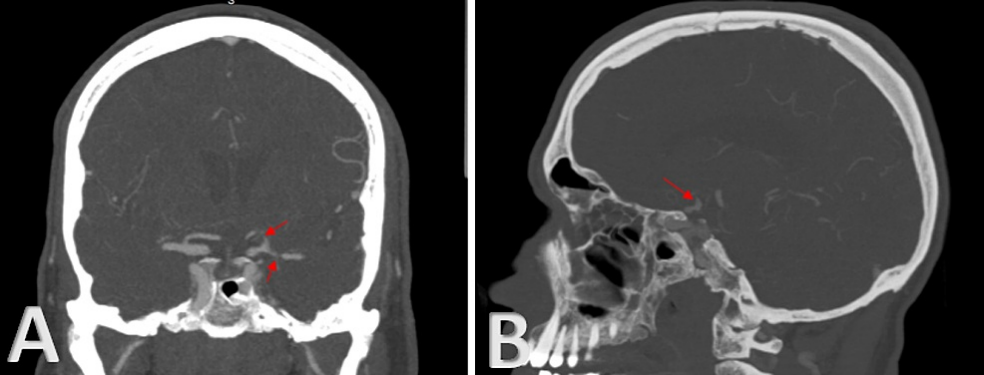
(A) Coronal view of CTA head demonstrates severe stenosis in left A1 and M1 (red arrows). (B) Sagittal view of CTA head demonstrates moderate stenosis in the left ICA terminus (red arrow). CTA: computed tomography angiography; ICA: internal carotid artery

Six months later, he presented again with similar symptoms of acute-onset right hemiplegia and dysphasia. MRI of the brain with and without contrast demonstrated a small acute ischemic infarct in the left precentral gyrus and a 2 cm mass-enhancing lesion in the left basal ganglia concerning for neoplasm (Figure 2). Repeat CTA of the head and neck demonstrated mild stenosis in the right internal carotid artery (ICA) terminus, right A1 segment of the anterior cerebral artery (ACA), and right M1 and M2 segments of the middle cerebral artery (MCA). It also showed moderate-to-severe stenosis in the left ICA terminus, left A1 segment of ACA, left M1 segment of MCA, and mild stenosis in the M2 segment of MCA (Figure 3). He underwent left basal ganglia biopsy for mass seen on MRI brain which showed brain parenchyma with marked reactive astrogliosis (glial fibrillary acidic protein), microglial activation (CD163), and mixed chronic lymphocytic and inflammatory cell infiltrates in parenchyma and blood vessel walls (CD3, CD20, MUM1). Special stains for herpes simplex type 1/2 and toxoplasma were negative. Congo red stain was negative for amyloid deposition. The constellation of features was reported to be nonspecific but in conjunction with imaging studies raised the possibility of vasculitis. The patient subsequently developed seizures and was started on levetiracetam. His condition improved and he was discharged home with outpatient stroke rehabilitation as he still had reduced muscle strength in the right upper extremity (3/5 grade) and right lower extremity (4/5 grade) and mild expressive dysphasia.

**Figure 4:**
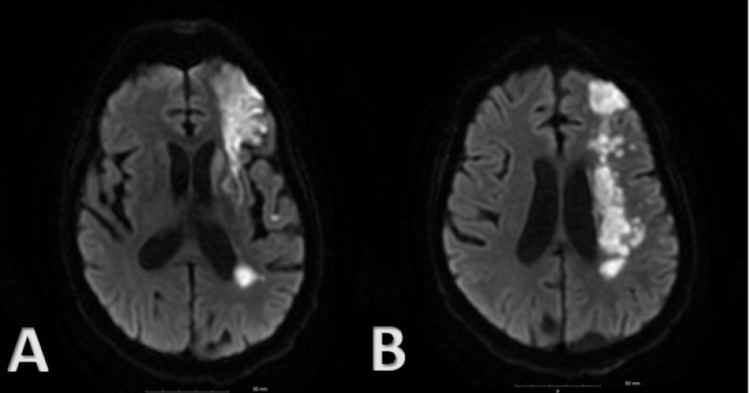
(A) MRI brain: DWI at the level of the basal ganglia. (B) MRI brain: DWI at the level higher than the basal ganglia demonstrating increased DWI signal in the left frontoparietal region consistent with acute watershed infarct. MRI: magnetic resonance imaging; DWI: diffusion-weighted imaging

**Figure 5:**
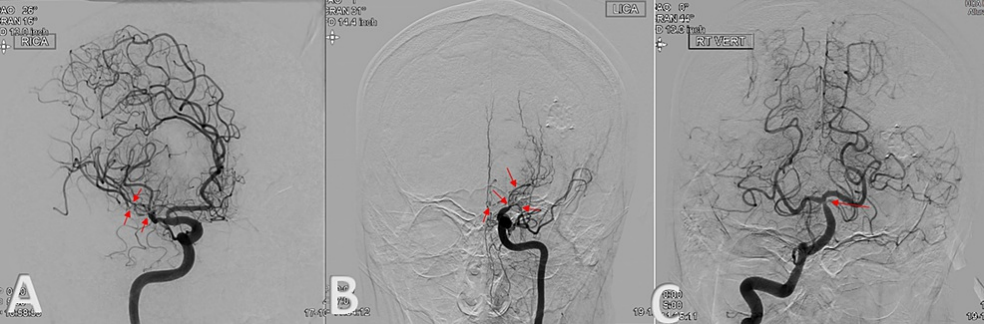
(A) Oblique view: right ICA angiogram demonstrating normal anterograde filling of the right ACA. Critical nearly occlusive stenosis of the right distal M1 MCA and proximal bilateral M2 MCAs, more severe in the inferior M2 MCA. (B) AP view: left ICA angiogram demonstrating critical occlusive stenosis of the supraclinoid ICA, faint filling of critically stenosed left ACA, and complete occlusion of the left MCA. Tangle tiny collateral vessels showing the characteristic appearance of “puff of smoke” consistent with Suzuki state V moyamoya syndrome. (C) AP view of the right angiogram demonstrating distal BA stenosis measuring approximately 25% per WASID criteria. Retrograde collateral filling of the bilateral MCA (mainly the left) as well as the left ACA territories. Left VA ends in PICA (not shown in this figure). ICA: internal carotid artery; ACA: anterior cerebral artery; MCA: middle cerebral artery; VA: vertebral artery; WASID: Warfarin-Aspirin Symptomatic Intracranial Disease; PICA: posterior inferior cerebellar artery

On the third hospitalization, one month later, he was admitted after suffering a seizure episode. On examination, he was found to have a new right facial droop, flaccid paralysis of both the right upper and lower extremities, and severe expressive dysphasia. Repeat MRI demonstrated increased diffusion-weighted imaging (DWI) signal in the left frontoparietal region consistent with watershed infarcts (Figure 4). Diagnostic cerebral angiogram demonstrated critical occlusive stenosis of the left supraclinoid ICA extending into the left A1 segment of the ACA, resulting in near-complete occlusion of the left ACA; complete occlusion of the left M1 segment of the MCA with a network of tangle tiny collateral vessels with the characteristic appearance of “puff of smoke;” and critical near-occlusion of the right distal M1 segment and proximal bilateral M2 segments of the MCA. These findings are consistent with Suzuki grade V moyamoya syndrome (Figure 5).

A secondary etiology was suspected after the patient presented with recurrent strokes despite the lack of risk factors, rapidly progressive stenosis seen on cerebral angiogram, as well as brain biopsy results suggesting possible vasculitis. Additionally, the patient was found to have multiple firm discrete violaceous plaques and nodules scattered over his chest, abdomen, arms, and legs concerning for Kaposi’s sarcoma. Sexual history was unobtainable from the patient due to severe expressive dysphasia. His family was unaware of any known diagnosis or source of infection for HIV or syphilis. Laboratory studies showed serum HIV RNA of 2,770,000 copies/mL, CD4 count of 37 (normal range: 359-1,519 uL), and initial total lymphocyte count of 400 cells/µL. Serum rapid plasma reagin titer was 1:2. Lumbar puncture analysis showed cerebrospinal fluid (CSF) venereal disease research laboratory test (VDRL) of 1:1, protein of 117 mg/dL (normal range: 12-60 mg/dL), glucose of 70 mg/dL (normal range: 40-70 mg/dL), and white blood count of 3 mm^3^ (normal range: 0-9 mm^3^). The patient was treated with intravenous penicillin G, aspirin, atorvastatin, levetiracetam, and atovaquone for *Pneumocystis* pneumonia prophylaxis. Transthoracic echocardiography was unremarkable. Antinuclear antibody, antineutrophil cytoplasmic antibody, and acute hepatitis panel were all negative. Erythrocyte sedimentation rate and C-reactive protein were initially elevated at 44 mm/hour (normal range: 0-20 mm/hour) and 77.9 mg/L (normal range: 0-9 mg/L), respectively, but trended down to normal after initiation of treatment. A jejunostomy tube was placed laparoscopically. The patient’s condition continued to deteriorate, and the palliative care team was consulted. The family subsequently elected for withdrawal of care, and the patient was discharged to the inpatient hospice where he expired shortly after.

This patient had a recurrence of strokes in the same MCA vascular territory over a relatively short seven-month time period, with rapid progressive changes on the CTA head with evidence of moyamoya syndrome with serum CSF evidence of *T. pallidum* and HIV/AIDS coinfection. This strongly suggests a rare diagnosis of rapidly progressing moyamoya syndrome secondary to vasculitis associated with a coinfection of AIDS and neurosyphilis.

## Discussion

Syphilis is a sexually transmitted infection that if left untreated, follows a course of active infection interrupted with periods of latent infection, making diagnosis challenging for the clinician. Despite the availability of early diagnostic tests and treatments, syphilis cases are continuing to rise in the United States [1,2]. The national rate of reported cases of primary and secondary syphilis in the United States increased from 2.1 cases per 100,000 in 2001 to 10.8 cases per 100,000 population in 2018 [1]. Neurosyphilis occurs with central nervous system involvement by *T. pallidum* infection. Definitive diagnosis is made with a reactive CSF-VDRL [2-4]. However, other findings include CSF lymphocytic pleocytosis and elevated protein concentration [3,4].

Many patients experience transient meningitis followed by spontaneous resolution. Clinical manifestations of persistent neurosyphilis vary and can be classified into early and late forms. Early forms of neurosyphilis are more commonly seen in HIV individuals [4,5]. These early forms consist of asymptomatic syphilis, acute aseptic meningitis, and meningovascular syphilis, whereas late forms present as dementia and tabes dorsalis with incontinence and sensory ataxia [4,5]. Individuals with low CD4 counts, detectable plasma HIV RNA, and with no antiretroviral therapy are more likely to have the early forms of neurosyphilis [5,6].

Patients with syphilitic meningitis may complain of typical symptoms of meningitis such as headache, confusion, nausea, and vomiting as well as neck stiffness [4,5]. Focal areas of inflammation that present as mass lesions contiguous with the leptomeninges are called syphilitic gummas. Cerebral syphilitic gummas on imaging can appear similar to brain tumors [7]. Postoperative pathology findings of one reported patient found to have a syphilitic gumma showed mixed inflammatory cell infiltration with sleeve-like infiltration of lymphocytes surrounding blood vessels in the outer layers of the gumma, suggestive of vasculitis [7]. We suspect that the 2 cm enhancing mass lesion seen in this patient could have been a syphilitic gumma as biopsy results are similar to this reported case. The lesion likely could have been the underlying cause of seizures in this patient as seizure episodes developed after mass was seen on imaging.

Meningovascular syphilis may present with focal neurologic deficits [2,4,5,8]. The underlying pathology is focal obliterative endarteritis with diffuse thickening and lymphocytic infiltration of perivascular spaces involving both medium and large-sized vessels (Heubner’s arteritis) as well as small intracranial vessels (Nissl-Alzheimer arteritis) [4,8]. The infectious arteritis may affect any vessel in the central nervous system leading to thrombosis, ischemia, and infarction [2,4,5,8]. The MCA and its branches are most commonly affected [8]. Moyamoya disease is a chronic progressive cerebrovascular stenosis of internal carotid arteries and arteries around the circle of Willis [9]. This chronic progressive stenosis predisposes patients to stroke [9]. Patients with characteristic moyamoya vasculopathy secondary to conditions such as meningitis, tuberculosis, syphilis, HIV, head irradiation, and sickle cell anemia are categorized as having moyamoya syndrome [9-12]. Ali and Gowda report a case of moyamoya syndrome in a 24-year-old patient found to have only meningovascular neurosyphilis [10]. Sharfstein et al. report a case of moyamoya syndrome in a 28-year-old woman with an eight-year history of AIDS on multiple antiretroviral medications and without associated neurosyphilis [11].

Moyamoya syndrome secondary to coinfection with AIDS and neurosyphilis has only been reported once in the literature by Morgello and Laufer [12]. In this case, a 22-year-old Haitian man presented with a 15-month course of progressive meningitis and recurrent cerebral infarcts. CSF findings including VDRL were unremarkable in the patient and he was presumed to have tuberculosis. Unlike our unique case, vessel imaging was not performed initially. Ten months after the presentation, a cerebral angiogram demonstrated findings consistent with moyamoya syndrome. In our case, the CTA head was unremarkable on presentation with significant progressive changes at six and seven months. Another difference between this reported case and ours is that it wasn’t until an autopsy was performed that diagnosis of quaternary meningovascular neurosyphilis and HIV coinfection was made. In our unique case, the diagnosis was made in the seventh month from his initial presentation. The rapidly progressive findings seen on CTA head and cerebral angiogram in our patient supports the hypothesis made by Morgello and Laufer that coinfection with *T. pallidum* and HIV may result in remarkably virulent forms of neurosyphilis [12].

Treatment of neurosyphilis is intravenous or intramuscular penicillin for 10-14 days [2-4,13]. Penicillin desensitization is preferred in patients with penicillin allergy, although ceftriaxone can be used if this is not possible [13]. Antiretroviral therapy is needed if HIV infection coexists. Patients should be followed up with a lumbar puncture and CSF white blood cell count and VDRL testing at intervals of three to six months to assess treatment efficacy, given that T. pallidum does not culture in vitro [2-4,13].

## Conclusions

Here, we report a unique case of rapidly progressive moyamoya syndrome secondary to meningovascular neurosyphilis in the setting of a late diagnosis of HIV/AIDS. This case highlights the importance of early diagnosis and treatment of HIV and syphilis given the severe complications of concomitant infection. It wasn’t until the recurrence of strokes in the same MCA vascular territory over a relatively short period of time in a patient with no significant risk factors that a secondary etiology was explored.
